# Surgical Management of Congenital Muscular Torticollis in a Child Using Z-plasty: A Case Report and Literature Review

**DOI:** 10.7759/cureus.112456

**Published:** 2026-07-11

**Authors:** Abdelouahid Taleuan, Mohammed ElAmine Squalli Houssaini, Adnane Lakhal, Abdellatif Oudidi

**Affiliations:** 1 Department of Otolaryngology - Head and Neck Surgery, Faculty of Medicine and Pharmacy, Al Amir Sultan Hospital, Errachidia, MAR; 2 Department of Otolaryngology - Head and Neck Surgery, Hassan II University Hospital, Faculty of Medicine and Pharmacy, Sidi Mohammed Ben Abdellah University, Fez, MAR

**Keywords:** congenital muscular torticollis, craniofacial asymmetry, sternocleidomastoid muscle, surgical release, z-plasty

## Abstract

Congenital muscular torticollis (CMT) is characterized by unilateral shortening of the sternocleidomastoid muscle (SCM), producing ipsilateral head tilt and contralateral rotation of the face and chin. It usually manifests during infancy and responds favorably to conservative treatment based on physiotherapy; spontaneous resolution is expected in the majority of cases. However, when diagnosis is delayed and patients present at a later age, conservative approaches tend to yield limited outcomes, neglected presentations remain prevalent in developing countries such as Morocco, where patients often present beyond the optimal age for conservative management.

We report the case of an 11-year-old female patient with CMT, who was successfully managed through unipolar SCM release combined with Z-plasty lengthening, yielding significant functional improvement and a satisfactory cosmetic outcome. This case underscores that surgical correction remains a viable and effective therapeutic option in older children with neglected CMT, even when conservative management has failed and the deformity has become chronic.

## Introduction

Congenital muscular torticollis (CMT) is an idiopathic condition manifesting in the neonatal period as a rotational and flexion deformity of the neck, arising from progressive shortening and fibrosis of the sternocleidomastoid muscle (SCM) [1,2]. It constitutes the third most common congenital musculoskeletal anomaly, following congenital hip dislocation and clubfoot [3].

When diagnosed early, CMT is amenable to conservative management with excellent outcomes using conservative physiotherapy, with spontaneous resolution expected in the majority of cases. The ideal timing for surgical intervention remains a matter of debate; nevertheless, in patients beyond one year of age, operative correction has been shown to confer both functional and aesthetic benefits, with the best outcomes being obtained between the ages of one and four years [4,5].

Beyond this window, neglected presentations pose a significant clinical challenge; the present report describes the surgical management of an 11-year-old female with longstanding untreated CMT, in whom unipolar SCM release combined with Z-plasty lengthening achieved significant functional and cosmetic improvement. Although early childhood remains the ideal window for surgical correction, this case illustrates that satisfactory functional and aesthetic outcomes are still attainable later in childhood, even when presentation has been delayed and the deformity has become chronic.

## Case presentation

An 11-year-old female child presented with the complaint of persistent neck stiffness and progressively restricted head movement. The pregnancy had been well attended with regular prenatal follow-up, and delivery was by uncomplicated vaginal route with no documented obstetric trauma. Since then, the patient’s attitude has been characterized by a tilting of the head to the right with a mild rise of the ipsilateral shoulder and the projection of the chin to the left side. No therapeutic intervention had been initiated during infancy or early childhood, and the deformity had persisted and progressively worsened in the complete absence of any rehabilitative or medical management.

**Figure 1:**
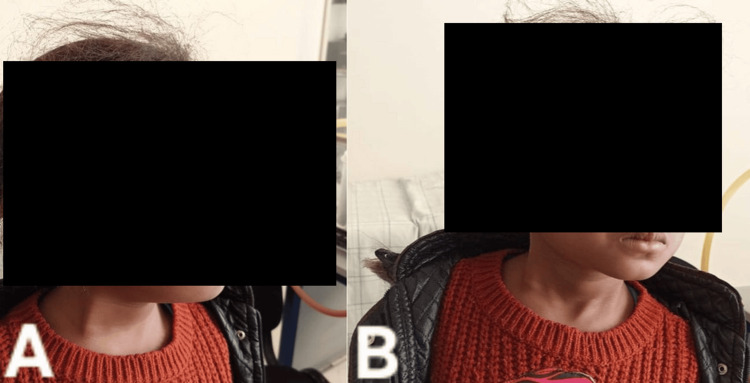
(A) Restricted neck rotation; (B) neck in a relaxed position.

On examination, restricted movements were observed. Facial asymmetry was evident, characterized by hypoplasia of the ipsilateral maxillary and mandibular prominences; mild elevation of the right shoulder was also noted (Figure 1). On palpation, no tenderness was noted. Neurological and ophthalmological examinations were unremarkable, excluding neurological and ocular causes of torticollis. Computed tomography (CT) scan of the cervical spine was normal, excluding osseous abnormalities.

Prior to surgical consideration, the patient was enrolled in an intensive physiotherapy program comprising 20 supervised sessions. After six months of physiotherapy, surgery was necessary because of a persistent head tilt and deficits of passive rotation.

**Figure 2:**
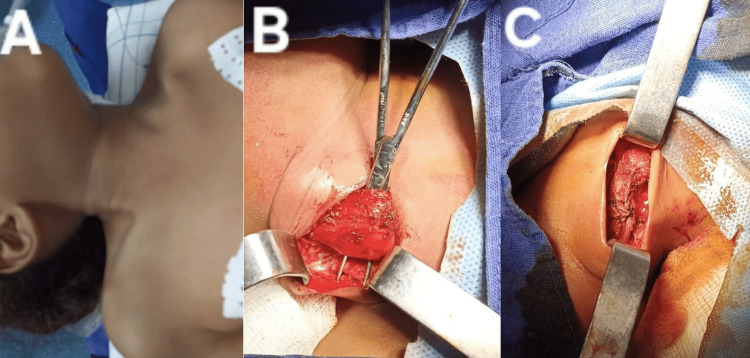
(A) Patient positioning for surgery; (B) exposure of the SCM; (C) Z-plasty lengthening of the SCM. SCM: sternocleidomastoid muscle

The procedure was performed under general anesthesia with the patient positioned supine. The head was rotated away from the operative side, while the neck was hyperextended and the chin directed toward the contralateral shoulder in order to place the affected side under tension (Figure 2). A horizontal incision was made 2 cm superior to the medial third of the clavicle. Dissection was carried through the platysma and the surrounding fascial and soft tissue layers, which were released. A unipolar release combined with Z-plasty lengthening of the right SCM was then performed using 3-0 absorbable monofilament sutures applied in a stretched position to maintain the cosmetic V-shape of the neck. Passive range of motion assessed intraoperatively immediately following release demonstrated a significant and immediate improvement.

Postoperatively, the patient was fitted with a soft cervical orthosis to be worn for eight hours per day over the first four weeks, maintaining the corrected head position and preventing reversion to the habitual deformity. This was followed by an intensive outpatient physiotherapy program of three supervised sessions per week for three months, followed by a home exercise regimen to be continued beyond the supervised rehabilitation phase.

**Figure 3:**
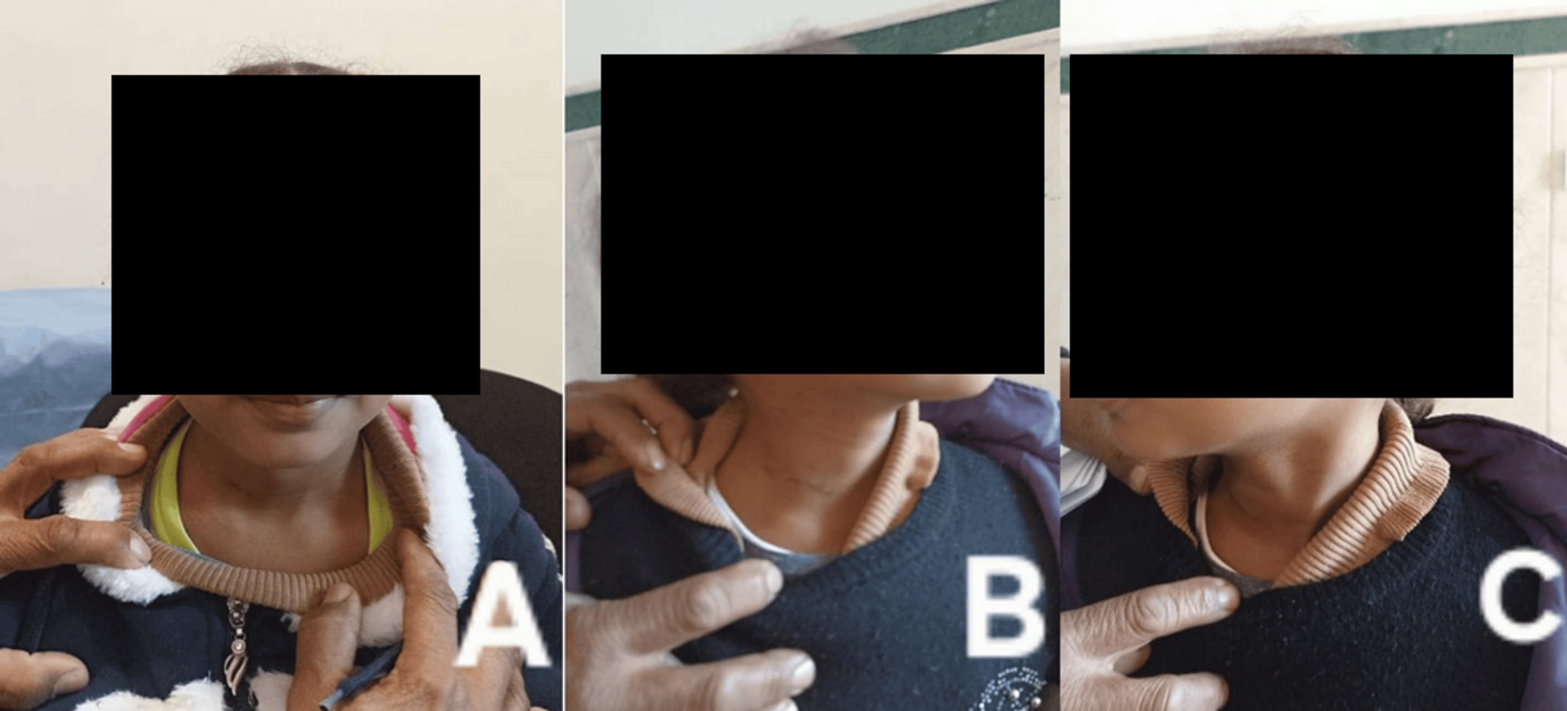
(A) Relaxed position with good correction of head tilt and chin deviation; (B) normal rotation on the left side; and (C) normal rotation on the right side.

An excellent result was noted, with complete restoration of cervical axial alignment to the midline, marked resolution of the head tilt, and marked improvement in active cervical range of motion in all directions. Chin deviation was no longer detectable on clinical assessment, and the patient and her family reported high satisfaction with both the functional and aesthetic results of the procedure, after an 18-month follow-up (Figure 3). No surgical complications were encountered during the entire follow-up period.

## Discussion

The most widely accepted pathophysiological mechanism involves intrauterine vascular compromise of the SCM, leading to compartment syndrome within the muscle and subsequent replacement of normal muscular architecture by fibrous tissue. Alternative etiopathogenic hypotheses that have been proposed include perinatal ischemia, hereditary predisposition, neurogenic causes, and infectious processes, although none have been definitively established as a primary mechanism [1].

Clinically, CMT manifests as contracture and shortening of the SCM muscle, resulting in ipsilateral head tilt and rotation of the face and chin toward the contralateral side. This abnormal cervical posture may give rise to positional plagiocephaly or facial asymmetry over time. Prolonged supine positioning further contributes to flattening of the contralateral aspect of the skull. When inadequately treated, fibrosis of the cervical musculature ensues, leading to progressive restriction of cervical mobility, craniofacial asymmetry, plagiocephaly, compensatory scoliosis, and elevation of the ipsilateral shoulder, all of which tend to worsen with advancing age [4,5].

The natural evolution of CMT is favorable in the majority of cases, with complete resolution occurring either spontaneously within the first months of life or following the early introduction of conservative measures, most notably controlled passive manual stretching. Such non-operative management is generally initiated before the age of one year. In this regard, Sönmez et al. [6] identified that approximately 95% of patients diagnosed and managed within the first year of life did not ultimately require surgical intervention. These findings are corroborated by Celayir [7], who similarly concluded that surgery can be avoided in early-treated patients, emphasizing that a successful outcome is closely tied to adequate parental compliance and cooperation throughout the rehabilitation process.

Nonoperative therapy beyond the age of one year is rarely successful, and operative correction becomes increasingly necessary. A study indicated that the ideal age for surgical intervention is between one and four years, when the tissues remain relatively flexible and secondary abnormalities have not yet become firmly set, thus enhancing the probability of favorable functional and aesthetic results. Nevertheless, several published case reports and case series suggest that surgery may still result in substantial improvement in adult patients with neglected CMT, even in cases where the deformity has become chronic [8]. Chen and Ko [9] reported that late SCM release performed in patients over six years of age could also give acceptable results. Another study involving 14 adolescents aged over 10 years likewise observed substantial enhancements in range of motion, tilt, and neck aesthetics in all subjects, with no postoperative complications reported [10]. While earlier intervention undoubtedly offers superior outcomes, late surgical correction can still yield clinically meaningful benefits and substantially improve quality of life, as the present case clearly illustrates.

The outcome observed in our patient is consistent with previous reports demonstrating that satisfactory functional and cosmetic results can be achieved in neglected CMT despite delayed presentation.

Several surgical techniques have been proposed for the treatment of CMT, including percutaneous and open tenotomies, unipolar release of the SCM muscle, and bipolar release with or without Z-plasty lengthening [9-11]. The main objectives of surgical intervention are to restore cervical range of motion, correct head posture, and achieve satisfactory cosmetic outcomes. Both unipolar and bipolar release have been reported to achieve satisfactory functional and cosmetic results in appropriately selected patients [9,10]. Compared with simple SCM release, Z-plasty provides controlled muscle lengthening while preserving the natural V-shaped contour of the neck, which may contribute to improved cosmetic outcomes without compromising functional correction [11,12]. Potential complications encountered after surgical release include recurrence, with rates of up to 7% after unipolar release that may require a secondary bipolar procedure [12]. Other reported complications include postoperative hematoma, hollowing at the base of the neck, bony prominence of the sternal head of the clavicle, and reattachment of the clavicular head resulting in a lateral fibrous band requiring further surgical release [12]. Compared with bipolar release, unipolar release has been associated with a shorter operative time, reduced blood loss, and avoidance of dissection around the upper end of the SCM, thereby minimizing the risk of injury to the greater auricular nerve [13]. In our patient, unipolar release combined with Z-plasty achieved complete correction without recurrence after 18 months of follow-up, suggesting that this technique may provide excellent outcomes even in selected neglected cases.

The postoperative period represents a critical phase of the overall management strategy, and preoperative head position is a pain-avoidance strategy, resulting in suboptimal adherence to the prescribed rehabilitation protocol. Failure to correct this posture risks progressive retightening of the previously released structures. Immobilization in an overcorrected position has been advocated as a means of optimizing surgical outcomes, particularly when combined with an intensive supervised physiotherapy program and a sustained long-term home exercise regimen [13].

## Conclusions

Early diagnosis and appropriate conservative management are generally successful in the majority of infants, while surgical treatment remains a safe and effective option for selected patients with persistent deformity after failed conservative therapy. Nevertheless, neglected presentations continue to constitute a significant clinical challenge in developing country settings, where delayed medical consultation remains commonplace.

Although surgical intervention is ideally performed during early childhood, our case demonstrates that excellent functional and cosmetic results can still be achieved in older children with neglected CMT despite delayed presentation and the chronicity of the deformity. Distal unipolar SCM release combined with Z-plasty lengthening proved to be a safe and effective procedure when combined with structured postoperative rehabilitation.
